# Clinical presentation and management of childhood intussusception in South Africa

**DOI:** 10.1007/s00383-021-04946-7

**Published:** 2021-07-02

**Authors:** Sharon Cox, Aletha Withers, Marion Arnold, Milind Chitnis, Corné de Vos, Mari Kirsten, Susanna M. le Grange, Jerome Loveland, Sello Machaea, Ashwini Maharaj, Shabir A. Madhi, Jacqueline E. Tate, Umesh D. Parashar, Michelle J. Groome

**Affiliations:** 1 Division of Paediatric Surgery, Red Cross War Memorial Children’s Hospital, University of Cape Town, 6th Floor ICH Building, Klipfontein Road, Rondebosch, Cape Town, 7700 South Africa; 2 Department of Paediatric Surgery, University of the Witwatersrand, Johannesburg, South Africa; 3 Tygerberg Hospital, University of Stellenbosch, Cape Town, South Africa; 4 East London Hospital Complex, Walter Sisulu University, East London, South Africa; 5 Steve Biko Academic Hospital/Kalafong Hospital, University of Pretoria, Pretoria, South Africa; 6 Universitas Hospital, University of the Free State, Bloemfontein, South Africa; 7 Inkosi Albert Luthuli Hospital, University of Kwa-Zulu Natal, Durban, South Africa; 8 South African Medical Research Council: Vaccines and Infectious Diseases Analytics Research Unit, Faculty of Health Sciences, University of the Witwatersrand, Johannesburg, South Africa; 9 Department of Science and Technology/National Research Foundation: Vaccine Preventable Diseases, University of the Witwatersrand, Johannesburg, South Africa; 10 Centers for Disease Control and Prevention, Atlanta, GA USA

**Keywords:** Intussusception, Reduction methods, Surgical intervention, Outcomes, Paediatric

## Abstract

**Purpose:**

We assessed management and outcomes for intussusception at nine academic hospitals in South Africa.

**Methods:**

Patients ≤ 3 years presenting with intussusception between September 2013 and December 2017 were prospectively enrolled at all sites. Additionally, patients presenting between July 2012 and August 2013 were retrospectively enrolled at one site. Demographics, clinical information, diagnostic modality, reduction methods, surgical intervention and outcomes were reviewed.

**Results:**

Four hundred seventy-six patients were enrolled, [54% males, median age 6.5 months (IQR 2.6–32.6)]. Vomiting (92%), bloody stool (91%), abdominal mass (57%), fever (32%) and a rectal mass (29%) represented advanced disease: median symptom duration was 3 days (IQR 1–4).

Initial reduction attempts included pneumatic reduction (66%) and upfront surgery (32%). The overall non-surgical reduction rate was 28% and enema perforation rate was 4%. Surgery occurred in 334 (70%), 68 (20%) patients had perforated bowel, bowel resection was required in 61%.

Complications included recurrence (2%) and nosocomial sepsis (4%). Length of stay (LOS) was significantly longer in patients who developed complications. Six patients died—a mortality rate of 1%. There was a significant difference in reduction rates, upfront surgery, bowel resection, LOS and mortality between centres with shorter symptom duration compared longer symptom duration.

**Conclusion:**

Delayed presentation was common and associated with low success for enema reduction, higher operative rates, higher rates of bowel resection and increased LOS. Improved primary health-care worker education and streamlining referral pathways might facilitate timely management.

## Introduction

Intussusception, occurring when one section of bowel invaginates into an adjoining distal section, is the most common cause of intestinal obstruction in children aged 3 months to 6 years, with patients younger than 3 years having a predominantly “idiopathic” aetiology. Males are affected more often [1–3], and up to 90% are ileocolic [4, 5]. Ultrasound combines advantages of high sensitivity and specificity with a lack of radiation and can accurately delineate the pathology [6]. The gold standard for non-surgical reduction remains the enema, either hydrostatic, or pneumatic, with reduction rates of 69% and 80% respectively, the preference amongst most centres in North America being pneumatic reduction (PR) [7, 8]. Current protocols allow for repeat attempts at enema reduction (ER) in selected initially unsuccessful cases. Operative management is required when non-operative reduction fails, if there is a pathological lead point, or when clinical findings suggest bowel ischemia, necrosis, perforation and may be required if there is established intestinal obstruction [9]. When management is delayed, congestion, ischemia, necrosis, and perforation may occur with death a possible consequence [5].

A recent review on intussusception in Africa of over 1100 patients reports a far from ideal situation [10] with late presentation of more than 3 days between symptom onset and treatment. ER rates are minimal and almost 90% of patients proceed to surgery. Reported mortality rates in African countries vary from 2% to 33.7% with a mean of 16% [10].

A retrospective study by Moore et al. describing clinical presentation, management and outcomes of intussusception in children conducted from 1998 to 2003 across 5 of the 9 provinces in South Africa [2], showed a 19% success rate of ER, with 40% of the cases proceeding to surgery requiring bowel resection. Subsequent to this publication, several paediatric surgical units made a concerted effort to increase the rate of PR and reduce surgical intervention through altering institutional management protocols [11]. Simultaneously, the number of paediatric surgeons has increased across the country. In addition, improvements in referral pathways, health systems and transport networks have occurred.

A prospective trial to assess intussusception risk after rotavirus immunization was conducted in South Africa from 2013 to 2017 [12]. Apart from the intussusception risk aspects, this study also collected information on presentation, diagnosis, reduction and surgical aspects of care for children with intussusception. This 5 year multi-site prospective observational study was conducted a decade after Moore’s study and included patients from the same five provinces. It aimed to assess presenting signs and symptoms, diagnosis, evaluation, management, and outcomes and compare these with previously published data to assess any improvements. In addition, it aimed to document seasonal occurrence, management techniques and outcomes across participating academic hospitals in South Africa.

## Methods

All patients age ≤ 3 years presenting with intussusception as defined by Level 1 Brighton Collaboration criteria [13], between September 2013 and December 2017, to nine academic hospitals across five provinces of South Africa were approached for enrolment in a prospective trial evaluating both intussusception risk following rotavirus vaccination [12] as well as clinical aspects of presentation and management of their intussusception. Participating hospitals included Charlotte Maxeke Academic and Chris Hani Baragwanath Academic (CHBAH) in Johannesburg, East London Hospital Complex (Frere and Cecilia Makiwane), Inkosi Albert Luthuli in Durban, Red Cross War Memorial Children's and Tygerberg Hospitals in Cape Town, Steve Biko Academic/Kalafong in Pretoria, and Universitas Hospital in Bloemfontein.

In addition, intussusception cases from July 2012 to August 2013 at CHBAH were retrospectively enrolled as this institution was already involved in a previously commenced study collecting the same clinical data. Approvals were obtained from the ethics committees of the Universities of Witwatersrand, Kwa-Zulu-Natal, Cape Town, Stellenbosch, Free State, Pretoria, and Walter Sisulu University. The study was registered on the South African National Clinical Trial Register (DOH-27-0913-4183). Written informed consent was obtained from the parent/guardian prior to enrolment of patients in the prospective part of the study.

Demographic characteristics, clinical signs and symptoms, methods of diagnosis, reduction methods, surgical intervention and patient outcomes were obtained by parent interview and hospital record review. All hospitals use similar pressure and duration protocol for reduction attempts whether pneumatic or hydrostatic. The protocols maintain sustained pressure via a rectally inserted Foley catheter with an inflated balloon, and buttock strapping at 80 mmHg for 1 min duration on 3 attempts, with a 1 min interval between each attempt, then proceeding to 100 mmHg and 120 mmHg sequentially for three times 1 min intervals at each pressure if needed and safe to do so. One hospital attempted ER in theatre under no sedation and proceeded directly to operation under general anaesthetic if not initially successful. All others would attempt a second ER between 4 and 6 h after the initial attempt if clinical circumstances were appropriate—no obvious perforation, no clinical deterioration, some degree of movement of the intussusceptum and a continued non peritonitic abdominal examination. In one hospital it was not always possible to perform an ER at night due to staffing constraints.

Statistical analysis was performed using Statistica (TIBCO Software Inc, Version 13.5.0.17) and Microsoft Excel for Microsoft 365 (Microsoft Corporation, Version16.01.13001.20266). Categorical variables were described as numbers and percentages. Non-parametric continuous variables were described using the median and inter-quartile range. Parametric continuous variables were described using the mean and standard deviation (SD). Inferential statistics for non-parametric variables, such as length of stay (LOS) and age of the patients, were performed using the Mann–Whitney *U* test (M–W *U* test). Regression analysis was performed to ascertain significance in the difference in presentation between seasons. A *p* value of < 0.05 was considered significant.

## Results

A total of 476 children, 258 (54%) males and 218 (46%) females, were enrolled in the study. The median age of participants was 6.5 months (2.6–32.6 months). Admission weight was recorded for 458 participants. Median weight was 7.5 kg (3.1–15.1 kg).

There were significantly more admissions during spring (September–November) 143 (30%) compared to other seasons [Summer 109 (23%), Autumn 112 (24%), and Winter 111(23%) (*p* = 0.03)].

Presenting symptoms included vomiting (92%), blood in the stool (91%), refusal to feed (66%) and diarrhoea (59%). The most common clinical signs were an abdominal mass (57%), fever (32%) and a rectal mass (31%) (Table 1). The median duration of symptoms prior to presentation at the study hospital was 3 days (IQR 1–4 days).

**Table 1:** Frequency of presenting signs and symptoms

Symptom/sign	Frequency *n* (%)
Symptoms	
Vomiting	436/475 (91.8)
Blood in stool	430/471 (91.3)
Refusal to feed	312/471 (66.2)
Diarrhoea	280/474 (59.1)
Clinical finding	
Abdominal mass	265/464 (57.1)
Fever	141/435 (32.1)
Rectal mass	138/447 (30.8)

Based on clinical signs and symptoms, and prior to radiological investigation, clinicians suspected the diagnosis of intussusception in 417/432 (97%) of patients, with different diagnoses suspected clinically in 15, and this data not recorded for the rest.

Ultrasound confirmed the diagnosis in 326/476 (69%) patients. Perfusion of the intussusceptum was assessed in 290 patients, confirmed in 132 (46%) and reported as absent in 78 (30%) patients. When analysed, ultrasound assessment did not correlate with outcomes—some indicating no perfusion reduced on the first PR attempt, whilst others that reported perfusion had necrotic bowel at operation. Other methods of diagnosis included contrast enema in 1 (0.2%) or CT scan in 5 (1%), and 62 (13%) were diagnosed at surgery.

**Fig. 1:**
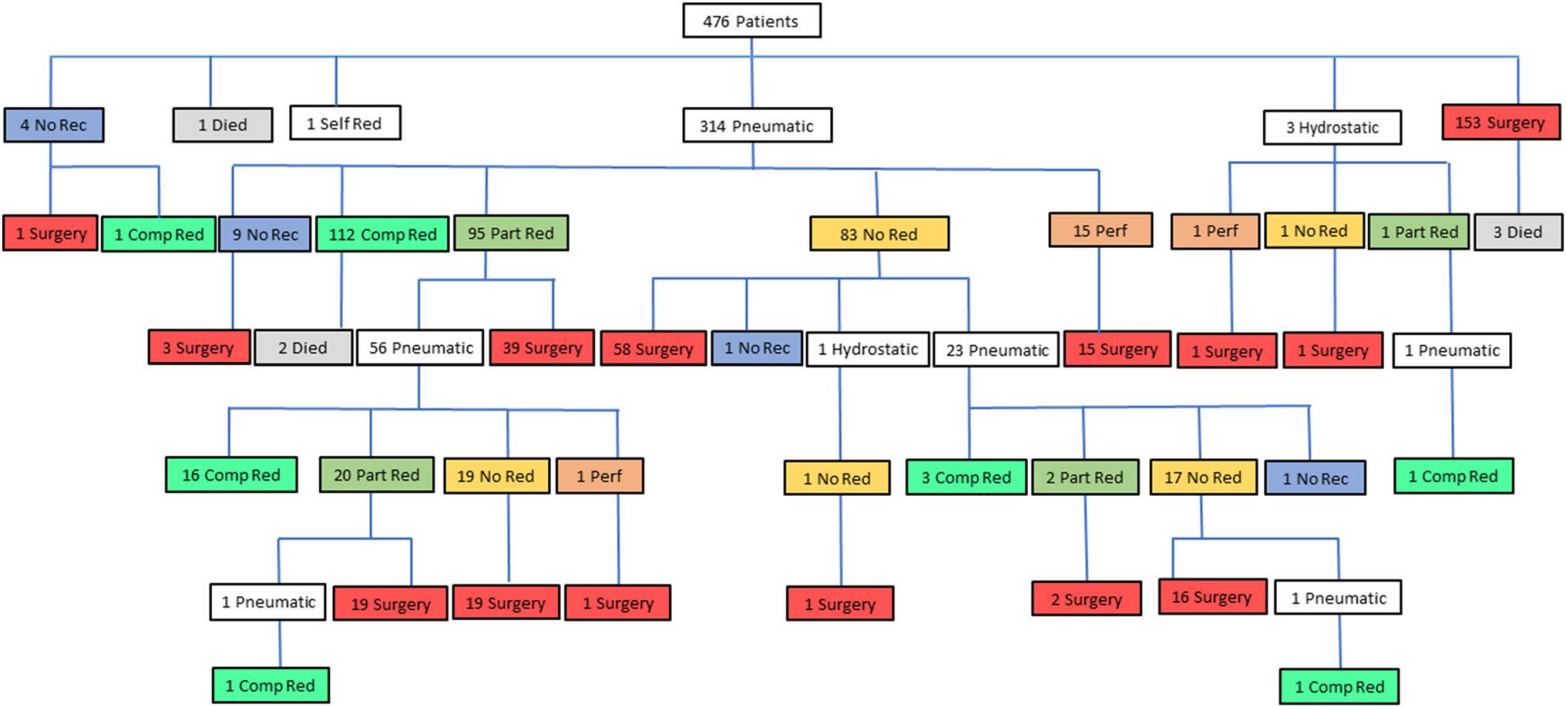
Flow chart indicating patient management pathways (*no rec* not recorded, *self red* self reduction, *pneumatic* pneumatic reduction attempt, *hydrostatic* hydrostatic reduction attempt, *comp red* complete reduction, *part red* partial reduction, *no red* no reduction, *perf* perforation)

Reduction methods were recorded for 470 (96%) patients (Fig. 1). PR was attempted in 314 (67%) patients. Complete reduction was achieved in 112/314 (36%), partial reduction in 95 (30%), no reduction in 83 (26%) and perforation occurred in 15 (5%) on the first attempt. Outcome was not documented in 9 (3%). A second attempt at PR was performed in 80 (25%) patients with no outcome recorded in 1 (1%), partial reduction in 22 (28%), complete reduction in 20 (25%), with 36 (45%) remaining unreduced and one further perforation (1%). A third attempt at PR was performed on two patients and was successful in both.

HR was attempted in three patients resulting in one perforation, one failed reduction (both progressed to surgery), and one partial reduction where a later attempt at PR was successful (Fig. 1).

A palpable rectal mass (the intussusceptum of ileocolic intussusception) was present in 138 (29%) patients, of which nine had prolapsed from the anus. Of these, 79 (57.3%), including four with prolapse, proceeded to PR. Complete reduction was achieved in 34 (43%) of these patients after 1 (27 patients) or 2 (7 patients) attempts at PR, and 8 (10%) perforated before or during the procedure.

Overall, there were 400 attempts at ER (in a total of 317 patients) including pneumatic and hydrostatic, and results were documented for 393 of these (Fig. 1). Successful ER occurred in 135 out of 400 reduction attempts, giving a reduction rate per procedure of 34% in those in whom any form of reduction was attempted. The overall reported non-surgical reduction rate in the whole cohort was thus 135 reductions (28%) out of 476 patients. The overall perforation rate at ER was 17 (4%) out of 400 reduction attempts.

Reduction pressures were recorded in 309/400 (77%) reduction attempts. The maximum pressures used at attempted reduction are documented in Table 2. Reduction was abandoned at lower pressures if the child did not tolerate the reduction, a perforation was noted, or the reduction was successful. During the first reduction attempt almost 65% of patients required pressures of 120 mmHg in an attempt to achieve reduction, and this increased to 80% during the second attempt.

**Table 2:** Maximal pressures used at attempted ER

Pressure (mm Hg)	Number of attempts (%)
Pressures used at 1st reduction (*n* = 244)	
≤ 80	50 (20.5)
≤ 100	36 (14.8)
≤ 120	158 (64.8)
Pressures used at 2nd reduction (*n* = 65)	
≤ 80	3 (4.7)
≤ 100	10 (15.6)
≤ 120	52 (79.7)

**Fig. 2:**
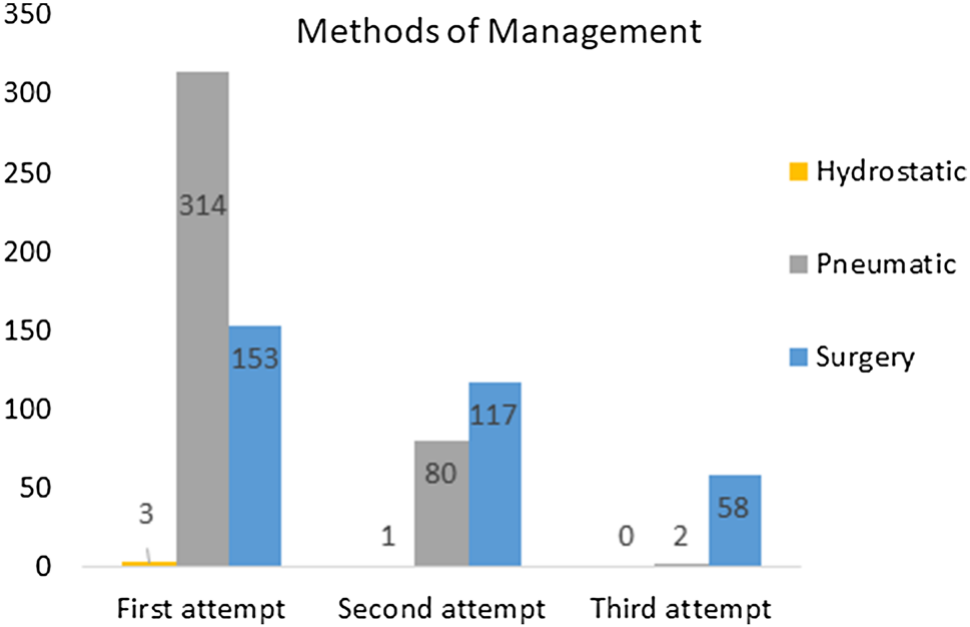
Sequential management attempts showing relative decrease in reduction attempts and increase need for surgery to treat intussusception

Surgical intervention was required in 334/476 (70%) of all patients. Of these, 153 (46%) progressed directly to surgery with no attempt at ER—initial surgery thus representing 32% of the entire cohort of 476 patients. Reasons for progressing directly to surgery included a peritonitic or markedly tender abdomen in 97 (63%) patients, 30 of whom had perforated and a further 67 of whom required resection of necrotic or ischaemic bowel. Other reasons for direct progression to surgery were when intussusception was not suspected, or patients were deemed unsuitable for ER due to co-morbid pathology or previous surgery. Reasons for direct progression to surgery was not recorded in 47 patients. A further 117 (35%) were treated surgically after a single attempt at ER and included 59 of those not reduced, 39 with partial reduction and 16 with perforations and 3 with an unrecorded initial outcome. An additional 58 (17%%) required surgery after further attempts at ER including 36 of those not reduced, 21 with partial reduction and the 1 that perforated. Figure 2 shows how, as management attempts progress, the relative contribution of surgery as a method of treatment increases and ER is performed less often.

With respect to surgical approach, laparotomy was performed in 323 (97%) patients, laparoscopy, and conversion to laparotomy in 8 (2%) and laparoscopy alone in 1, with 2 operative approaches not recorded. At operation, 68 (20%) patients had perforated bowel, and in 9 (3%) the intussusception had already reduced. Resection and anastomosis were required in 188 (56%), resection and stoma creation in 16 (5%) and 119 (36%) underwent manual reduction without surgical resection. The operative procedure was not recorded in 11 patients. Of those undergoing manual reduction, 51 (43%) proceeded directly to surgery while 68 (57%) had an initial, and a further 30 (25%) had a second attempt at PR. At total of 27 relook laparotomies were required in 26 (6%) patients.

Of patients undergoing surgery, those requiring resection had a significantly longer duration of symptoms prior to presentation than those not requiring resection (M–W *U* test, *p* = 0.012). In addition, the M–W *U* test comparing the overall need for resection vs successful reduction, whether PR, HR or surgical reduction without resection, showed a significantly shorter duration of symptoms than those requiring resection, *p* < 0.0001.

The location of the intussusception was documented in 405 patients: 380 (93%) ileocolic, 15 (4%) ileo-ileal and 10 (2%) colo-colic. A lead point was identified in 10 (2%) patients, and these were characterized as Meckel’s diverticulum in six and a polyp in four. Three of these presented with the intussusceptum palpable in the rectum. Seven underwent an attempt at initial pneumatic reduction, with partial or no reduction as a result, with a second attempt at pneumatic reduction in four; however all 10 progressed to surgery where it was found that 3 had perforated, and all required resection and anastomosis. Three lead point cases were ileo-ileal while the remainder occurred in the ileo-caecal region.

Including perforations found at ER, 67 (14%) patients experienced a total of 95 complications (excluding death). Of those, 10 (2%) recurred after PR. Five of these had successful repeat PR of the recurrence, the others required surgery. The most frequent complication was the development of nosocomial sepsis, occurring in 16 (3%) patients. Surgical complications occurred in 30 (9%) of 334 patients undergoing surgery. Complications ranged from wound sepsis in 7 (2%) to more severe complications including anastomotic leak in 5 (1%), post-operative bowel perforation in 3 (0.6%) and wound dehiscence in 3 (0.6%). Table 3 provides an overview of complications as well as their Clavien–Dindo grade.

**Table 3:** All complications as per the Clavien–Dindo classification and interventions [14]

Clavien–Dindo grade	Complication category	Number (%) *n* = 476	Overall total per Clavien–Dindo grade
Grade 1	Post-op ileus—treated conservatively	4 (0.8)	8 (1.7)
	Acute kidney injury, resolved with intravenous fluids	3 (0.6)	
	Refeeding syndrome	1 (0.2)	
Grade 2	Nosocomial sepsis	16 (3.4)	26 (5.5)
	Wound sepsis	7 (1.5)	
	Blood transfusions	2 (0.4)	
	Short bowel syndrome—managed with parenteral nutrition	1 (0.2)	
Grade 3a	Pneumothorax required drain	2 (0.4)	4 (0.8)
	Wound abscess—drained in ward	1 (0.2)	
	Entero-cutaneous fistula—managed conservatively	1 (0.2)	
Grade 3b	Perforation (at ER)	16 (3.4)	42 (8.8)
	Recurrence of intussusception	10 (2.1)	
	Anastomotic breakdown/leak	5 (1.1)	
	Perforations (post-operative, requiring surgery)	3 (0.6)	
	Wound dehiscence requiring relook	3 (0.6)	
	Negative relook for suspected perforation	2 (0.4)	
	Intra-abdominal collections	1 (0.2)	
	Necrotic stoma	1 (0.2)	
	Adhesive bowel obstruction	1 (0.2)	
Grade 4	Convulsions	3 (0.6)	9 (1.9)
	Hypoxia requiring CPR	2 (0.4)	
	Prolonged invasive respiratory support	2 (0.4)	
	Liver failure	1 (0.2)	
	Cardia arrest	1 (0.2)	
Grade 5	Died before treatment	2 (0.4)	6 (1.3)
	Death due to multi organ failure, sepsis	4 (0.8)	

The median duration of hospitalisation was 5 days (IQR 3‒7, 0–106 days). Patients who died had a significantly longer duration of symptoms before presentation than survivors (M–W *U* test, *p* = 0.022). LOS was significantly longer in patients who developed complications (*p* value < 0.0001). Six patients died giving a mortality rate of 1%. One of the babies died within hours of arrival at the hospital. The patient was 6 months old, severely dehydrated and shocked, and did not respond to resuscitation. The intussusceptum was presenting at the anus. One 6 month and one 2.5 month old baby underwent pneumatic reduction after initial stabilization, but died days later due to ongoing sepsis. The other three were 3, 4 and 5 months old respectively. All underwent surgery and required bowel resection and anastomosis. Two required a relook laparotomy, one for anastomotic breakdown and the other for further bowel and stoma necrosis. All were shocked and septic on admission, required admission to the intensive care unit, and died of overwhelming sepsis.

Outcomes including overall non-operative reduction rate, surgical, resection and perforation rates, length of symptom duration and LOS were stratified by participating hospital (Table 4). The three hospitals with shorter median duration and range of symptoms had a higher overall non-operative reduction rate and a lower overall operative rate, as well as no mortalities in their cohort of patients.

**Table 4:** Reduction, surgery, and resection rates by hospital listed in order from highest to lowest enema reduction rates

	Number of patients	Median symptom duration, days (IQR)	Overall non-operative reduction *n* (%)	Overall surgery *n* (%)	Perforated at operation *n* (%)	Resection performed *n* (%)	Median LOS, days (range)	Death *n* (%)
Site 5	56	2 (1–3)	38 (69.1)	17 (30.9)	3 (5.5)	7 (12.7)	1 (0–5)	0
Site 3	43	2 (1–3)	19 (44.2)	24 (55.8)	5 (11.6)	18 (41.9)	5 (1–60)	0
Site 8	54	2 (1–4)	21 (38.9)	33 (61.1)	12 (22.2)	18 (33.3)	4 (1–66)	0
Site 1	119	3 (2–4)	21 (17.8)	97 (82.2)	21 (17.8)	60 (50.8)	7.7 (0–51)	0
Site 4	92	3 (2–4)	14 (15.4)	77 (84.6)	8 (8.8)	49 (53.8)	6 (1–63)	3 (3.3)
Site 6	60	3 (2–4.5)	9 (15.0)	51 (85.0)	14 (23.3)	28 (46.7)	5 (0–65)	0
Site 2	29	3 (2–4)	3 (10.3)	17 (85.0)	4 (5.9)	12 (60.0)	6 (0–28)	2 (6.8)
Site 7	23	3 (1–5)	2 (9.1)	20 (90.9)	1 (4.5)	12 (54.5)	5 (0–106)	1 (4.3)

The difference in median LOS amongst the sites was statistically significant (*p* < 0.001). Site one and seven had a longer LOS compared to site five (*p* = 0.030 and *p* = 0.0256 respectively). Hospitals recording deaths had a longer median LOS of 6.00 days (IQR 4–9) compared to hospitals with no deaths where a median LOS of 4.00 days was recorded (IQR 0.66), *p* < 0.001.

## Discussion

Many paediatric conditions present with vomiting and diarrhoea. The diagnosis of surgical conditions, including intussusception, is often delayed until additional signs or symptoms such as abdominal pain, distension, a palpable mass or blood per rectum occur [5]. Intussusception is a surgical emergency. Delay in presentation, diagnosis or management may result in significant morbidity and possible mortality. This study has highlighted these circumstances. The delayed presentation in most patients likely led to poor rates of successful ER, high operative and resection rates as well as mortalities. To improve outcomes, patients require urgent referral, rapid transport to a management facility, and emergent management to avoid bowel congestion, ischemia, perforation and ultimate surgical resection [5].

The median age of patients was 6.5 months, correlating with global and African data where more than 50% of children present before age 1 [4, 5, 10]. Consistent with international literature, this study shows a male predominance [1, 5, 10].

Despite international studies not demonstrating seasonal variability [4], presentation was significantly more in the Spring months of September–November. This parallels findings in previous regional studies [2, 5] but does not correlate with the peak incidence of gastroenteritis admissions across the country [14, 15]. Traditional teaching is that rates of intussusception parallel rates of acute gastroenteritis—with gastroenteritis-causing lymphoid hyperplasia that acts as the lead point. Diarrhoea was only present in 60% of patients in this study. This outcomes trial was part of a prospective study of rotavirus vaccination outcomes and did not show a significant risk of intussusception after rotavirus vaccination [12]. Adenovirus infection is a known association with intussusception [5, 16, 17], and additional analyses are underway to evaluate enteric pathogens detected in this cohort from stool samples collected at the time.

Vomiting and bloody stools tend to occur later in the pathogenesis of intussusception [5], and as the disease becomes established progressive gaseous distension diminishes the ability to palpate a ‘sausage-shaped’ mass [9]. Over 90% of patients presented with vomiting and bloody stools, while 60% presented with a palpable mass, and in almost 30% the intussusceptum was palpable on rectal examination or presenting at the anus. Thus, clinical indications are that this cohort presented late, supported by an average duration of symptoms of 3 days. This is consistent with reports from Africa [10], but longer than typically presented in first world literature [18]. Reasons for late presentation were not analysed, but what is known is that many patients need to travel long distances, potentially from rural areas and most patients seeking public health care have limited access to transport. A longer symptom duration allows time for stasis, bacterial translocation and development of intestinal obstruction, all of which lead to significant metabolic anomalies, septicaemia, and dehydration. These need to be addressed with sometimes significant volume resuscitation and antibiotics prior to definitive management [9, 11]. Volume resuscitation, intravenous antibiotics as well as nasogastric tube drainage are requested by the receiving hospital on referral, however, the majority of patients are not discussed prior to presentation—many arriving with private transport from home as opposed to coming from primary or secondary care facilities, thus delaying initiation of therapy.

In this series, the diagnosis was clinically suspected in 97% of patients and was confirmed in most by ultrasound. This contrasts to other centres in Africa where diagnostic facilities and personnel may not be readily available, and where diagnosis remains largely clinical or surgical [10]. Ultrasound is the most reliable diagnostic tool with a sensitivity of over 98% and a specificity of 88–100%. It is valuable in defining location, detecting perfusion, diagnosing lead points, or confirming an alternative diagnosis [3, 16, 19]. Absence of blood flow on ultrasound is correlated with ischemia and necrosis, unsuccessful ER and the need for resection at operation [9, 20]. However, we found no correlation between ultrasound assessment of perfusion and reducibility or viability of the bowel, and thus would suggest in our setting to attempt ER in all patients where the clinical findings allow, as opposed to proceeding directly to theatre on the basis of perfusion findings.

Previously, a symptom duration of over 24–48 h was considered a relative contraindication to ER [9]. While this is no longer the case, patients with a longer history do have a reduced success rate for ER. Despite the 3 day median duration of symptoms in this series, over two-thirds of patients had one or more attempts at PR, indicating that local protocols have been updated and followed. In addition, some centres consider a rectal presentation of the intussusception as a contraindication to ER. Over 40% of those with a rectally palpable or prolapsed intussusception who were subjected to PR had a successful reduction indicating that ER should be attempted in these patients.

In a recent meta-analysis of over 32,000 children, PR and HR had an 82.7% and 66.6% reduction rate respectively [8]. In another study including 12,370 patients, surgery was only required in 1046, giving an 84% success rate of PR [3]. Our series only had three cases of HR attempted, and two proceeded to surgery. For patients undergoing ER, the initial success rate in this group was just over 35%. A second reduction, with a published success rate of about 50%, and thought to be one of the most important advances in management to reduce operation rates [9], yielded a low success rate of just 25%, giving an overall ER rate of only 28% of the 476 patients. This is a slight improvement on the 19% reported 15 years ago in the same region [2], but remains unacceptably low given the increased morbidity and costs related to operative reduction [9].

Assessment of reduction pressures confirmed adequate technique with almost 65% of patients in the first, and 80% in the second attempt receiving pressures of up to 120 mmHg in an attempt to achieve reduction. Either successful reduction, or perforation were the reasons for not reaching 120 mmHg.

Bowel perforation is a possible complication of non-surgical reduction. Both HR and PR techniques reportedly have similar perforation rates of about 0.4–0.8% [8, 16]. In contrast we demonstrated a perforation rate of 4%. This may be an indication of the high number of patients presenting with advanced disease. Perforation is usually pre-existing, either occurring secondary to a necrotic intussusceptum, or more commonly due to pressure necrosis and perforation in the distal colon which is unmasked by the reduction attempt.

Surgical consultation is recommended before any attempt at ER to assess for signs of peritoneal irritation that may indicate bowel ischemia or perforation [16]. Internationally surgery rates stand between 2% and 10% [3, 17] and theoretically, if ER protocols include extended indications and repeat reduction attempts, only those cases requiring resection should proceed to surgery [9]. When attempting to understand why 70% of our patients required surgical intervention, it is prudent to assess the route toward surgery and the surgical findings. Almost half of those undergoing surgery progressed directly to surgery as they were considered unsuitable for ER due to their clinical situation, most protocols dictating that patients with peritonitis, free air, established bowel obstruction and haemodynamically unstable patients require surgery [11, 21], or the service was not available at the time of presentation. Just over a third of patients had either perforated or were not reduced at ER and went on to surgery after the first attempt at ER. A further 17% required surgery after further attempts at ER. The high incidence of perforation as an operative finding, and the fact that resection bowel was required in two-thirds of patients operated, as well as the need for ileostomy in 5% of cases suggests that this cohort of patients are presenting with more advanced disease than those in international series. Indeed, other local studies confirm high rates of resection [2, 11]. In contrast, just over one-third of patients undergoing surgical intervention required no resection, only manual reduction. Analysis of these patients reveals that almost 60% had an initial, and a further 25% a second attempt at ER prior to proceeding to theatre. There are thoughts that procedurally correct enema reductions should avoid this [9], yet, the attempted reductions in these patients appear to have been performed according to protocol. The other 40% of patients who had manual reduction at operation were taken directly to operation with no attempt at ER. While under resuscitation and inappropriately delayed surgery do contribute to higher morbidity and mortality rates, some of these patients were likely stable and well resuscitated and could have been treated with non-operative reduction. Even if this were the case, however, the rate of surgery in this cohort would still be close to 60%.

While most cases of idiopathic intussusception occur in the ileo-caecal region, other anatomical sites are generally related to a lead point [3, 4]. Higher rates of colo-colic intussusception have been reported in African children [22, 23]. While ileocolic intussusceptions predominated, just over 30 patients (7%) had intussusception at other sites, including 3 ileo-ileal cases secondary to a Meckel’s diverticulum.

There is a higher incidence of lead points in older children, with younger children tending to present with idiopathic causes (5). Lead points occur between 1.5% and 12% of cases [5], with a 2% incidence in this series. This is a relatively low incidence but speaks to the inclusion criteria of a maximum age of 3 years. Consistent with the literature, most lead points were discovered at operation [3], after one or two attempts at PR—the diagnostic ultrasound having failed to detect the lead point. In younger children, commonly reported lead points are Meckel’s diverticulum and duplication cysts [24], but these occurred infrequently in this series, and may be an indication of the infective aetiology in patients in this low-middle income country setting.

Reported recurrence rates after PR or HR reduction vary from 6 to 10% [8, 16]. This series showed a recurrence rate after initial successful PR to be comparable with international literature at 7.3%. Repeat PR is effective [24], confirmed in this series where 50% were successfully re-reduced pneumatically. There were no recurrences after surgical intervention—but this does have a reported rate of about 1% [16]. Apart from perforations and recurrence, most complications experienced relate to their poor general condition and the propensity for sepsis, with nosocomial and wound sepsis being the most frequent, despite prophylactic antibiotics being a standard part of the protocol.

The mortality rate of 1% in this study is an improvement on previously published studies in this country that reported rates between 2 and 7% [2, 11], and a vast improvement on mortality rates reported across Africa [10]. In addition, it is nearing the reported mortality rates of < 1% in developed countries where patients present earlier.

When analysing the data from different sites, the sites with the shorter duration of symptoms have a higher overall non-operative reduction rate and a lower overall operative rate. These same sites with a median duration of symptoms of 2 days also had no mortalities in their cohort of patients. Once again, analysis of data relating to symptom duration is significant in indicating that late presentation results in further disease progression, an increased requirement for surgery and a poorer outcome [11].

Increased surgical rates result in increased LOS. There was a significant difference in LOS between Site five (with the highest rate of non-operative reduction) and other sites. Essentially the data show that increased duration of symptoms trends toward increased operative rate, which is associated with increased morbidity and mortality.

When analysing the entire cohort of patients and comparing to the study conducted 15 years earlier in the same group of hospitals, it is taking longer from onset of symptoms for patients to arrive at tertiary care institutions, and yet, the non-operative reduction rate has increased from 19 to 28%. In addition, 20% more of those going to theatre require resection of bowel. Despite this, the mortality rate across the centres has dropped. All of this indicates improved adherence to ER protocols and improved intra-hospital management.

After full analysis of all the data in this report it is apparent that late presentation and its consequences, both anatomically and physiologically, is the single most important factor contributing to the high surgical intervention and morbidity rate. While some steps can be taken within the academic hospitals across the country to improve outcomes, increasing awareness of health workers in district hospitals and rural health facilities, and improvements in referral and transport are required.

Our study had some limitations. Due to problems with early recognition of intussusception and transport of patients from rural areas, we may have missed patient deaths prior to reaching the hospital. Some patients may have been managed at secondary level hospitals by general surgeons and would thus not be included in the data. While all hospitals have a similar protocol as relates to pressures and timing of reductions, there were a few differences: one institution reported the lack of late-night reduction services, and another institution performs their reductions in theatre and then proceeds to operation immediately if the reduction is unsuccessful, thus negating the possibility of a second attempt. Another limitation is that the reason for progressing directly to theatre was not recorded in 10% of the study population. Knowing this would give more information as relates to how services could be improved. This study only represented five of the nine provinces in South Africa but we believe that the hospitals and areas from which patients were enrolled are representative of the current situation in the whole country. Despite this being predominantly a prospective study, some of the case records were not fully completed.

## Conclusion

This study includes 476 patients with intussusception, 90% under 12 months of age, with a slight male predominance, presenting to 9 tertiary care institutions across 5 provinces of South Africa. The patients are presenting 3 days (IQR 1–4) after symptom onset with the consequences of more advanced disease, decreased ER rates, high operative rates with over 40% of the entire cohort needing bowel resections. The improved ER and mortality rates compared to previous studies are testament to the improvement in-hospital care these patients receive.

Despite the centres included in this study adhering to recent advances in intussusception reduction, including improved and internationally acceptable non-operative reduction protocols, broadening the indications for non-operative reduction, and the use of repeated enemas, this study, while showing minimal improvement in the last 10 years, has not been able to emulate international norms. While the number of Paediatric surgeons in the country slowly climbs, it is clear that South Africa needs a better infrastructure, referral networks and improved patient transport to improve outcomes. The significant differences in outcomes demonstrated in this study between different units in terms of duration of symptoms and associated impact on management options and morbidity and mortality emphasize this. Improved population-level as well as primary care health-care worker education and streamlining of referral pathways from primary health-care settings is needed to increase early diagnosis and achieve outcomes reflected in high income country series.
